# Controlled trial of cervical cancer screening frequency among human‐papillomavirus‐vaccinated women

**DOI:** 10.1002/ijc.70229

**Published:** 2025-11-07

**Authors:** Mònica Ortega Llobet, Penelope Gray, Iacopo Baussano, K. Miriam Elfström, Tiina Eriksson, Camilla Lagheden, Pekka Nieminen, Anna Söderlund‐Strand, Joakim Dillner, Ville N. Pimenoff, Matti Lehtinen

**Affiliations:** ^1^ Centre for Cervical Cancer Elimination, Department of Clinical Science, Intervention and Technology Karolinska Institutet Stockholm Sweden; ^2^ International Agency for Research on Cancer Lyon France; ^3^ Medical Faculty Tampere University Tampere Finland; ^4^ Department of Obstetrics and Gynecology University of Helsinki Helsinki Finland; ^5^ Department of Laboratory Medicine, Clinical Microbiology Skåne University Hospital Lund, Lund University Lund Sweden; ^6^ Research Unit of Population Health, Faculty of Medicine Oulu University Oulu Finland; ^7^ Finnish Institute for Health and Welfare Department of Vaccines Helsinki Finland

**Keywords:** cervical screening, HPV‐vaccination, RCT

## Abstract

Cervical screening frequency has not been studied in vaccinees. As the major risk factor, oncogenic human papillomavirus (HPV) is declining due to vaccination. We report a trial to assess the effectiveness of cervical screening frequency among women HPV‐vaccinated as early adolescents (NCT02149030). In 2013, 5626 1992‐1995‐born women, who had received three doses of the HPV16/18 vaccine at ages 12–15 between 2007 and 2010 in a community‐randomized vaccination trial (NCT00534638), were allocated at age 22 into high‐intensity cytology‐based cervical screening by even birth date (Arm A1) or into low‐intensity cytology‐based cervical screening by odd birth date (Arm A2). One thousand three hundred thirty‐three women who received HPV16/18 vaccination at age 18 attended a safety of low intensity‐screening arm (Arm A3). Low‐intensity screening, where low‐grade cytological abnormalities were not revealed for 6 years, was compared to the standard high‐intensity screening used in Finland at the time. The prevalence of cytological and HPV findings was calculated at ages 22/25/28. The hazard ratio of histopathologically confirmed immediate cervical cancer precursors (HSIL/CIN2+) among participants was compared between low‐ and high‐intensity screening arms. The overall occurrence of CIN2+ was comparable in Arms A1, 0.70% and A2, 0.66%, with the corresponding hazard ratio at age 28 being 0.97 (95% confidence intervals, 0.50–1.88). By age 28, the occurrence of vaccine‐HPV types 16/18 was reduced up to 88% in the 12‐to‐15 compared to 18‐year‐old HPV‐vaccinated women. In conclusion, the risk of CIN2+ was similar for HPV‐vaccinated women who attended low‐intensity cervical screening compared to high‐intensity screening most likely due to the decline of oncogenic HPVs.

## Graphical abstract

What's new?

Since the introduction of the human papillomavirus (HPV) vaccine in 2006, vaccine‐type infections have declined markedly. As nonvaccine HPV types generally carry lower oncogenic potential, this shift warrants reevaluation of cervical screening regimens. Here, associations between different cervical screening intensities and high‐grade squamous intraepithelial lesion (HSIL) occurrence were explored among Finnish women vaccinated against HPV in early adolescence. HSIL cases were nearly equivalent between high‐ and low‐intensity screening groups. Vaccine‐targeted HPV16/18 and cross‐protected HPV31/45 were infrequent, with no detection in HSIL lesions. The findings support less intensive cervical screening for HPV‐vaccinated women to better balance screening risks and benefits.

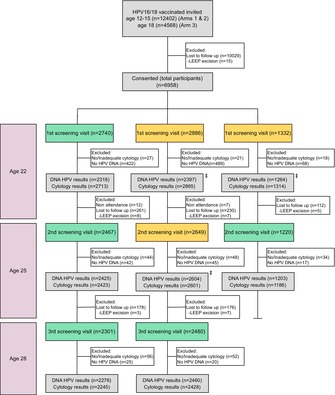

**Abbreviations**
ASCUS: Atypical Squamous Cells of Undetermined Significance
CIN2+: Cervical Intraepithelial Neoplasia grade 2+
HPV: Human Papillomavirus
HSIL: High‐grade Squamous cell Intraepithelial Lesion
LSIL: Low‐grade Squamous cell Intraepithelial Lesion
PPV: Positive Predictive Value

## 1 INTRODUCTION

Cervical cancer is one of the most common cancers in women globally.^1^ Implementation of cervical cancer screening and subsequent diagnosis and treatment of identified precancerous cervical lesions has proven to be one of the most cost‐effective preventive measures against a human cancer to date.

Licensed prophylactic human papillomavirus (HPV) vaccines^2^, ^3^ are highly effective against cervical neoplasia and have been implemented in the national vaccination programs of many countries worldwide.^4^, ^5^ This has resulted in a drastic decline in the circulation of the most oncogenic HPV infections and associated cervical lesions in vaccinated birth cohorts, most notably in countries such as Scotland which has achieved over 90% vaccination coverage through school‐based vaccination programs.^4^, ^6^ In Denmark and Sweden, vaccination against HPV6/11/16/18 was launched more than 10 years ago, resulting in a continuous decrease in cervical intraepithelial neoplasia grade 2+ (CIN2+).^7^, ^8^ In Finland, school‐based vaccination against HPV16/18 of early adolescents was implemented by means of a community‐randomized trial in 2007–2010 targeting both girls and boys born in 1992–1995. With 52% vaccination coverage this resulted in a 90% prevalence reduction of HPV types 16 and 18 in less than 10 years.^5^, ^9^

In many countries HPV‐vaccinated birth cohorts have now entered cervical screening programs which are based on HPV testing due to its overall better performance.^10^ Reductions in the positive predictive value (PPV) of cervical cytological screening and the specificity of HPV‐DNA testing following HPV vaccination have now been confirmed.^11^, ^12^ This is due to both vaccine‐induced direct protection and herd effect against the most common, vaccine‐covered high‐risk HPV types the prevalence of which both among the vaccinated and unvaccinated women has significantly declined.^13^, ^14^, ^15^

Recent studies have reported evidence of lower oncogenicity HPV types replacing vaccine‐covered HPV types 16/18 (31/45), which may further impact the positive predictive value of HPV‐based screening for non‐16/18 high‐risk HPV.^16^ Furthermore, considering the lower oncogenic potential of infections caused by non‐vaccine targeted HPV types, there is a risk for unnecessary referrals to colposcopy and overtreatment of lesions which would otherwise regress, thus impacting the balance of benefit versus harm. This indicates that the intensity of cervical screening among HPV vaccinated birth cohorts needs to be revisited.^12^, ^15^

There is no controlled trial evidence on the effect of low screening intensity and risk of CIN2+, particularly not among women who received HPV vaccination as early adolescents. We conducted a systematic allocation trial of high intensity versus low intensity cytology‐based cervical screening (cytology was still an option when our trial was launched in 2014) among women aged 22 to 28 years old (NCT02149030), who had received three doses of the bivalent HPV16/18 vaccine between ages 12 and 15.^17^

## 2 METHODS

### 2.1 Study design and procedures

In 2007, all residents of 33 Finnish communities born in 1992–1995 were invited to participate in a community randomized vaccination trial (NCT00534638).^17^ This encompassed 33 out of the 34 Finnish communities with more than 35,000 inhabitants (outside of the Capital Metropolitan area). Virtually all (99.4%) female participants received HPV vaccination with three doses of the bivalent HPV16/18 vaccine either at the age of 12 to 15 (12,402 participants), or, in the case of the trial participants who had earlier received hepatitis B virus vaccination, a delayed HPV vaccination at the age of 18 (4568 participants). In total, 16,988 HPV16/18‐vaccinated participants were invited to attend the screening trial at the age of 22.

Women HPV‐vaccinated at the ages 12 to 15 were individually allocated into high‐intensity (Arm A1, individuals with even birth dates) and low‐intensity (Arm A2, individuals with odd birth dates) screening arms. They were invited to attend three screening visits with a 3‐year interval at the ages of 22, 25, and 28. Women HPV‐vaccinated at age 18 were invited to a parallel low‐intensity screening safety arm (Arm 3) until the age of 25 for the early assessment of the safety of low‐intensity screening in an interim analysis.^17^ In total, 6958 women consented to participate.

Participants in the high‐intensity screening arm, A1, received information on all cytological findings at each screening visit while participants in the low‐intensity screening arm, A2, only received information on cytological findings in the case of high‐grade squamous intraepithelial lesion (HSIL), atypical squamous cells, cannot exclude HSIL or atypical glandular cells that indicated referral to colposcopy according to the local standard of care. For arm A2, other cytological results were not revealed to the providers, and no action was taken on low‐grade cytology results in the low‐intensity screening arm until the end of the trial at the age of 28 years old, and in the case of the safety arm A3, at the age of 25 years old.

Participation in the study ended if the loop electrosurgical excision procedure was performed during the study.

**FIGURE 1:**
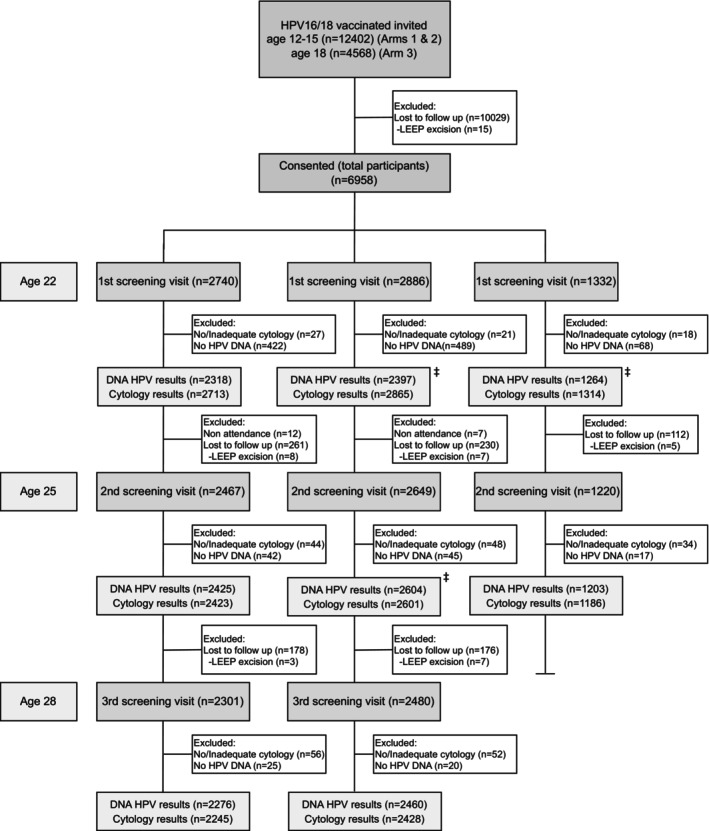
Flowchart of the study design and participation throughout the three study visits. Participants missing both ages 25 and 28 visits were considered lost to follow‐up. Participants in arm A2 at age 22, and 25 and in arm A3 at age 22 (‡), were blinded to the results of cytology visits unless these showed a case of HSIL, ASC‐H, or AGC. AGC, atypical glandular cells; ASC‐H, atypical squamous cells, cannot exclude HSIL, HSIL, high grade squamous cell intraepithelial lesion.

All arm A1 and A2 participants received information on cytological findings at the end of the trial. HPV DNA findings were disclosed at the end of the study. Participants in arm A3 attended screening visits at the ages of 22 and 25 but received full cytological information at age 25. The study outline and participation by arms is illustrated in a flowchart (Figure 1) and Appendix S1.

Self‐collected cervicovaginal samples for HPV DNA testing and clinician‐taken samples for cytological pap‐staining were obtained at all consecutive screening visits. The participants were also asked to answer a health and sexual behavior questionnaire at the first screening visit at the age of 22.

### 2.2 Laboratory analyses: HPV‐genotyping

All cervical samples were HPV‐genotyped using matrix‐assisted laser desorption time of flight mass spectrometry, Luminex^18^, ^19^ and/or the BD Onclarity™,^20^ platforms for the detection of high‐risk HPV types 16, 18, 31, 45, 51, 52 and HPV types 33/58, 56/59/66 and 35/39/68 compiled into pools.

### 2.3 Statistical analysis

The hazard ratios of histopathologically confirmed cervical intraepithelial neoplasia grade 2+ findings in the low‐intensity screening arm versus the high‐intensity screening arm (A2/A1) were calculated using binomial regression with a complementary log–log link function. Survival analysis and one minus Kaplan–Meier curves were used to plot the cumulative hazards and respective risk table for the main outcome.

Non‐inferiority of overall screening effectiveness conveyed by the low‐intensity screening in women at age 28 years compared to that conveyed by high‐intensity screening in women at ages 22, 25, and 28 was demonstrated if the difference between the two groups in the upper limits of the 95% confidence intervals (95% CIs) for their CIN2+ prevalence estimates was below 10%.^21^

All statistical analyses were conducted in R Studio (version 2023.06.2) using the following packages: tidyverse (v. 2.0.0), data.table (v. 1.15.4), and plyr (v. 1.8.9) for descriptive statistics; Epi (v. 2.51), Survival (v. 3.8‐3), survminer (v. 0.5.0), and Caret (v. 6.0–94) for calculations of prevalence, hazard ratios and prevalence ratios; and ggplot2 (v. 3.5.1) for graphical visualization.

## 3 RESULTS

In 2014, all 16,988 female participants of the Finnish community‐randomized HPV vaccination trial were invited to participate in a study on the effectiveness of low‐intensity versus high‐intensity screening. Almost all (99.4%) of these women had received three doses of HPV16/18 vaccine either between ages 12 and 15 (arms A1/A2, 12,402 women) or at age 18 (arm A3, 4586 women). Respectively, 5626 (45.4%) and 1332 (29.0%) of the invited women, consented to participate between 2014 and 2017 at age 22. The former were systematically allocated into high‐intensity (arm A1) or low‐intensity (arm A2) cervical screening at ages 22, 25, and 28. Arm A1 participants received all cytological information of samples taken at all study visits; Arm A2 participants received cytological information only on HSIL+ lesions at the study visits. All cytological information was disclosed to arm A2 participants at age 28 when the study ended (Figure 1). Arm A3 comprised women who had received the HPV16/18 vaccination at age 18.

Our trial achieved a moderate rate of enrollment (5626/12,402 eligible women, 45.4%) among the women who had received HPV16/18 vaccination as early adolescents. However, the participants showed high compliance (4778/5626, 84.9%) throughout all three screening visits. Eight hundred and forty‐eight women were lost to follow‐up due to a change of contact information and/or a change of residence.

The baseline characteristics between these two arms were comparable. In the high‐intensity screening arm, A1, 28% of women (631/2255) reported current smoking, compared to 28% (665/2372) women among the low‐intensity screening arm, A2. The average sexual debut age was reported to be 16 years old (16.42 in arm A1, 16.50 in arm A2 and 16.57 in arm A3) in all arms (Table 1).

**TABLE 1:** Baseline characteristics of women, who received HPV16/18 vaccine at age 12–15 (A1/A2) or 18 (A3), and were randomized to high‐intensity cervical screening at ages 22, 25, and 28 (A1), or low‐intensity screening at age 28 (A2) or at age 25 (A3 safety arm).

Variable	A1 (*N* = 2740)^a^	A2 (*N* = 2886)^b^	A3 (*N* = 1332)
	*n*/mean (%/SD)	*n*/mean (%/SD)	*n*/mean (%/SD)
Total questionnaire answers	2255 (100)	2372 (100)	1047 (100)
Missing from questionnaire	489	510	286
Average age of sexual debut	16.42 (1.88)	16.50 (1.85)	16.57 (1.82)
Average age of HPV‐vaccination	14.34 (0.69)	14.34 (0.71)	18.74 (0.49)
Number of lifetime partners			
0	93 (0.04)	90 (0.04)	38 (0.04)
1	369 (0.16)	371 (0.16)	200 (0.19)
2	256 (0.11)	278 (0.12)	132 (0.13)
3	272 (0.12)	284 (0.12)	121 (0.12)
4	256 (0.11)	295 (0.12)	125 (0.12)
5 or more	950 (0.42)	1001 (0.42)	397 (0.38)
Missing	59 (0.03)	53 (0.02)	34 (0.03)
Smoking			
Never	1325 (0.59)	1409 (0.59)	681 (0.65)
Quit	271 (0.12)	266 (0.11)	112 (0.11)
Current	631 (0.28)	665 (0.28)	244 (0.23)
Other than cigarettes	21 (0.01)	19 (0.01)	4 (0.003)
Alcohol intoxication			
Never	507 (0.22)	528 (0.22)	248 (0.24)
Less than once a month	1442 (0.64)	1494 (0.63)	656 (0.63)
1–2 times a month	276 (0.12)	316 (0.13)	125 (0.12)
More than once a week	23 (0.01)	23 (0.01)	14 (0.01)
Missing	7 (0.003)	11 (0.005)	4 (0.004)
Condom use			
Always	420 (0.19)	483 (0.20)	203 (0.19)
Beginning of a relationship	693 (0.31)	749 (0.32)	341 (0.33)
Sporadic relationship	720 (0.32)	774 (0.33)	340 (0.32)
After forgetting the pill	362 (0.16)	385 (0.16)	179 (0.17)
Never	73 (0.03)	78 (0.03)	28 (0.03)
Missing	757 (0.16)	735 (0.16)	345 (0.17)

^a^ Frequent (A1).

^b^ Infrequent (A2).

Overall, the prevalence of cytological findings did not differ significantly by screening arm (Table 2). We observed a moderate, albeit non‐significant increase in the prevalence of atypical squamous cells of undetermined significance (ASCUS) lesions at all ages in the low screening intensity arm A2 as compared to the high screening intensity arm A1 (prevalence ratio of 1.17%, 95% CI 1.00–1.37). The prevalence of low‐grade squamous intraepithelial lesion (LSIL) fluctuated slightly by arm and study visit. The prevalence of HSIL was noticeably stable at the start and end of the screening for both arms A1 (0.18–0.33%) and A2 (0.21–0.27%). The overall prevalence of HSIL in the low‐intensity screening arm (19/2865, 0.66%) was not inferior to that of the high‐intensity screening arm (19/2713, 0.70%) (Table 2).

**TABLE 2:** Prevalence with 95% confidence intervals (95% CIs) of cytological findings (ASCUS/LSIL/HSIL) in women, who received the HPV16/18 vaccine at age 14 and were randomized to high‐intensity cervical screening (A1), or low‐intensity screening (A2), and prevalence ratios (with 95% CI) of the identified cytological findings at ages 22, 25, 28 and combined.

Age	Arm	ASCUS cases *n* (*N*)	Prevalence (%) (95% CI)	Prevalence ratio (95% CI)
22	A1	102 (2713)	3.76 (3.04–4.47)	1.10 (0.85–1.43)
	A2	119 (2865)	4.16 (3.43–4.89)	
25	A1	86 (2423)	3.55 (2.81–4.29)	1.23 (0.94–1.62)
	A2	114 (2601)	4.39 (3.60–5.17)	
28	A1	81 (2245)	3.61 (2.84–4.38)	1.14 (0.86–1.52)
	A2	100 (2428)	4.12 (3.33–4.91)	
All 22–28	A1	253 (2713)	9.32 (8.23–10.4)	1.17 (1.00–1.37)
	A2	313 (2865)	10.9 (9.78–12.1)	

Age	Arm	LSIL cases *n* (*N*)	Prevalence (%) (95% CI)	Prevalence ratio (95% CI)
22	A1	44 (2713)	1.62 (1.14–2.09)	0.65 (0.41–1.02)
	A2	30 (2865)	1.05 (0.67–1.42)	
25	A1	22 (2423)	0.91 (0.53–1.29)	0.97 (0.54–1.74)
	A2	23 (2601)	0.88 (0.52–1.24)	
28	A1	19 (2245)	0.85 (0.47–1.23)	0.88 (0.46–1.66)
	A2	18 (2428)	0.74 (0.40–1.08)	
All 22–28	A1	85 (2713)	3.13 (2.48–3.79)	0.78 (0.57–1.07)
	A2	70 (2865)	2.44 (1.88–3.01)	

Age	Arm	HSIL cases *n* (*N*)	Prevalence (%) (95% CI)	Prevalence ratio (95% CI)
22	A1	7 (2713)	0.26 (0.07–0.45)	0.95 (0.33–2.70)
	A2	7 (2865)	0.24 (0.06–0.43)	
25	A1	8 (2423)	0.33 (0.10–0.56)	0.82 (0.30–2.24)
	A2	7 (2601)	0.27 (0.00–0.47)	
28	A1	4 (2245)	0.18 (0.00–0.35)	1.16 (0.31–4.30)
	A2	5 (2428)	0.21 (0.03–0.39)	
All 22–28	A1	19 (2713)	0.70 (0.39–1.01)	0.95 (0.50–1.79)
	A2	19 (2865)	0.66 (0.37–0.96)	

The point prevalence of vaccine‐targeted HPV16/18 was remarkably low at the first study visit (HPV16 prevalence, *p* = 0.26% and 0.21% in arms 1 and 2 respectively, while *p* = 2.30% in arm A3) (Table 3). HPV18 was extremely rare in both arms A1 and A2 throughout the three screening visits (prevalence ranging 0.04%–0.13%), closely followed by HPV16 (prevalence ranging 0.20%–0.35%) (Table 3). The prevalence of vaccine‐covered HPV types 31 and 45 was decreased but higher than HPV16 and 18 at the first screening visit (respectively, P_HPV31_ = 0.56% and P_HPV45_ = 0.47% in arm A1 and P_HPV31_ = 0.71% and P_HPV45_ = 0.66% in arm A2). They continued to decrease at the following visits by ages 25 (respectively, P_HPV31_ = 0.29% and P_HPV45_ = 0.16% in A1; P_HPV31_ = 0.46% and P_HPV45_ = 0.27% in A2) and 28 (respectively, P_HPV31_ = 0.22% and P_HPV45_ = 0.13% in A1; P_HPV31_ = 0.20% and P_HPV45_ = 0.28% in A2).

**TABLE 3:** Type‐specific HPV DNA prevalence findings by age in women, who received the HPV16/18 vaccine at age 14 and were randomized to high‐intensity cervical screening at ages 22, 25, and 28 (A1), or low‐intensity screening at age 28 (A2) or age 25 (A3).

Age 22	A1 (*N* = 2318)	A2 (*N* = 2397)	A3 (*N* = 1264)
HPV type	*n* (%)	*n* (%)	*n* (%)
HPV16	6 (0.26)	5 (0.21)	29 (2.30)
HPV18	3 (0.13)	2 (0.08)	8 (0.63)
HPV31	13 (0.56)	17 (0.71)	26 (2.06)
HPV45	11 (0.47)	16 (0.66)	15 (1.19)
HPV51	180 (7.76)	174 (7.26)	89 (7.04)
HPV52	119 (5.13)	117 (4.89)	78 (6.17)
HPV33/58	94 (4.05)	118 (4.92)	72 (5.70)
HPV56/59/66	255 (11.0)	286 (11.93)	138 (10.9)
HPV35/39/68	167 (7.20)	151 (6.30)	82 (6.49)
Total hrHPV	696 (30.0)	724 (30.2)	393 (31.1)

Age 25	A1 (*N* = 2425)	A2 (*N* = 2604)	A3 (*N* = 1203)
HPV type	*n* (%)	*n* (%)	*n* (%)
HPV16	6 (0.25)	7 (0.27)	17 (1.41)
HPV18	2 (0.08)	2 (0.08)	2 (0.17)
HPV31	7 (0.29)	12 (0.46)	18 (1.50)
HPV45	4 (0.16)	7 (0.27)	3 (0.25)
HPV51	75 (3.09)	90 (3.46)	42 (3.49)
HPV52	100 (4.12)	123 (4.72)	42 (3.49)
HPV33/58	60 (2.47)	96 (3.69)	42 (3.49)
HPV56/59/66	169 (6.97)	201 (7.71)	95 (7.89)
HPV35/39/68	125 (5.15)	111 (4.26)	65 (5.40)
Total hrHPV	731 (30.1)	839 (32.2)	247 (20.5)

Age 28	A1 (*N* = 2276)	A2 (*N* = 2460)
HPV type	*n* (%)	*n* (%)
HPV16	8 (0.35)	5 (0.20)
HPV18	1 (0.04)	1 (0.04)
HPV31	5 (0.22)	5 (0.20)
HPV45	3 (0.13)	7 (0.28)
HPV51	49 (2.15)	49 (1.99)
HPV52	59 (2.59)	72 (2.93)
HPV33/58	44 (1.93)	63 (2.56)
HPV56/59/66	113 (4.96)	121 (4.92)
HPV35/39/68	75 (3.30)	86 (3.50)
Total hrHPV	291 (12.8)	323 (13.1)

The overall prevalence of non‐vaccine high‐risk HPV was high in the three arms. The most common type was HPV51 (P_HPV51_ = 7.76%, 3.09%, and 2.15% in A1, P_HPV51_ = 7.26%, 3.46%, and 1.99% in A2 and P_HPV51_ = 7.04% and 3.49% in A3 at the consecutive study visits, respectively) (Table 3). HPV52 was also common (P_HPV52_ = 5.13%, 4.12%, and 2.59% in A1, P_HPV52_ = 4.89%, 4.72%, and 2.93% in A2 and P_HPV52_ = 6.17% and 3.49% in A3 at the consecutive study visits, respectively). It is noteworthy that the prevalence differences of the non‐vaccine‐HPV types between the arms A1 and A2 and the safety arm A3 were smaller than those of the vaccine‐covered types (HPV16/18/31/45) (Table 3).

Fifty‐five cases of histologically confirmed cervical HSIL/CIN2+ were found during the 6 years of follow‐up between ages 22 and 28 in the arms A1 and A2. Of these 52, 18 were confirmed CIN3 cases (Table 4). The HPV genotyping revealed that none of these cases were positive for HPV16, 18 or 31, and only one case in arm A1 was positive for HPV45.

**TABLE 4:** Follow‐up findings in HPV16/18 vaccinated women with histologically confirmed high‐grade squamous intraepithelial neoplasia (CIN2+) participating in the high‐intensity (A1) or low‐intensity (A2) screened arms.

Arm	Genotyping				Baseline results age 18	
	Age 22	Age 25	Age 28	Diagnosis/year	Cytology	HPV DNA
A1	45, 51			CIN 2/2014	ASCUS	
A1	Negative	P1, P2, 52	P1, P2	HSIL, CIN 3/2020	Negative	Negative
A2	P1	P1, P2	P1	CIN 2–3/2020	Negative	Negative
A2	P1, 52	P2, 52	P1	CIN 2/2021	Negative	Negative
A1	52	52		CIN 2–3/2018	Negative	Negative
A2				CIN 2/2015	Negative	Negative
A2	52	P1, 52	52	HSIL, CIN 2/2022	Negative	Negative
A2		51	Negative	HSIL (outside of study) /2020	Negative	Negative
A2	P3			HSIL/	LSIL	66
A1	11			CIN 2/2015	LSIL	11,39
A2	P2	P2, P3		HSIL, CIN 2–3/2019		
A1	P2			CIN2 (outside of study)/2017		
A2	Negative			HSIL (outside of study)/2018		
A2	51	51	Negative	CIN 2, HSIL/2019	Negative	Negative
A1	51			CIN 3/2016		
A1	P1	P1, P3		Exocervix: LSIL, vagina: HSIL, VAIN3/2023		33,39,51,58
A2	P1, P3, 51	P1, 6, 52		CIN 3/2020		
A2	Negative	51	P2	CIN 2, HSIL/2019	Negative	Negative
A1	P2, 52			HSIL/2018	Negative	Negative
A1		P2		CIN 3/2019	Negative	66
A2	51		51	HSIL, CIN 3/2022		
A2	Negative	P2, 52	P1, P2, 52	HSIL, CIN 3/2022	Negative	Negative
A1	Negative	Negative		HSIL/2020	Negative	Negative
A1	P1			CIN 3/2020	ASCUS	33
A1	P1			HSIL/2018	Negative	Negative
A2	Negative	Negative	Negative	CIN 2–3/2019	Negative	Negative
A1	P2, 51, 52	P2, 51	Negative	HSIL, CIN 3/2020	Negative	Negative
A1	P2	P1, 87		HSIL/2020	Negative	Negative
A2	52	52	52	HSIL/	Negative	Negative
A1	52	52	52	HSIL, CIN 3/2023	Negative	Negative
A1	Negative	P2	Negative	HSIL, CIN 2/2022		
A1	P1			CIN 2/2019		56,66
A2		Negative		HSIL/2015	Negative	Negative
A2	P3	P1		HSIL/2018	Negative	Negative
A1	P1	Negative	Negative	CIN 2/2014		
A1	P2, 51	P2, 52, 61, 70		HSIL/2018		
A2	P1, P2, P3, 39	P1, 52		CIN 3/	Negative	6, 52
A1		51	51	HSIL/2020		
A2	P1			CIN 3/2015		
A2	P2, P3	P3	P3	HSIL, CIN 2–3/2021	Negative	Negative
A1	P1, P3			CIN 3/2015		
A1	P1	P1, P2		CIN 2/2021	Negative	51,56
A1	51	87, 90	Negative	CIN 2/2016	Negative	6
A2	Negative	52	P2, 52	CIN 1–2/	LSIL	6,56,58,66
A2	P3, 11, 51	P3	Negative	CIN2, HSIL/2020	Negative	Negative
A1	P3			CIN 3/2016	LSIL	35
A2	Negative			CIN 3 /2016	Negative	58
A1	P2	P3, 52, 89		HSIL/2022	Negative	Negative
A1	Negative	52	Negative	HSIL/2021		
A1	P1	P1		HSIL/2021		
A2	P1, P2, P3, 51			HSIL/2018		
A1		Negative	Negative	HSIL (outside of study)/2020		Negative
A2		P1	P1	CIN2, CIN3/2023		
A2		P1, P3	P1, P3	HSIL/2023	Negative	Negative
A2	P1	P1	P1	HSIL/2023	Negative	51, 52

*Note*: P1 = 33/58, P2 = 56/59/66, P3 = 35/39/68.

Almost half of these cases (*n* = 25/55) presented with at least a 3‐year persistence of hrHPV infections throughout the different study visits (9 in arm A1 and 16 in arm A2). The most common persistent hrHPV infections were caused by non‐vaccine types HPV33/58 (10 cases, 4 in arm A1 and 6 in arm A2), HPV51 (4 cases, 2 in arm A1 and 2 in arm A2) and HPV52 (7 cases, 2 in arm A1 and 5 in arm A2). Multiple infections were frequently seen, especially with HPV52.

**FIGURE 2:**
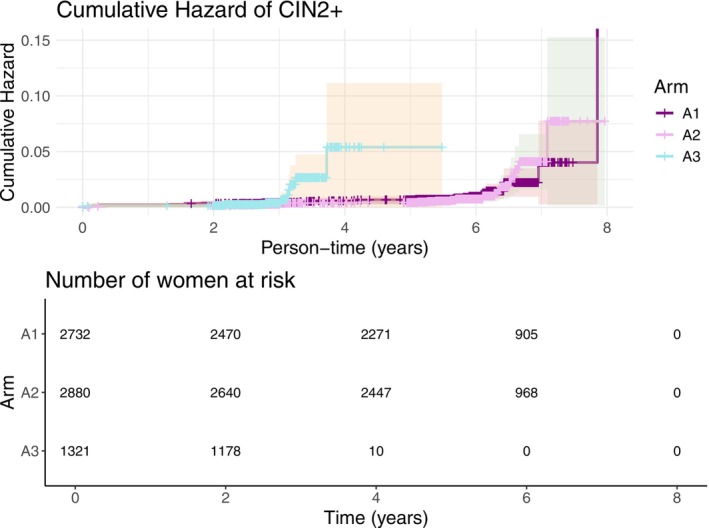
One minus Kaplan Meier showing the cumulative incidence of CIN2+ and risk table for frequent (A1), infrequent (A2) and safety (A3) arms, for all participants who attended all study visits as per protocol. Each participant's person‐time started at the date of their study visit at 22 years old and finished at their visit at 28 years old or when diagnosed with the outcome.

The hazard ratio of histologically confirmed HSIL/CIN2+ among the women participating in the low screening intensity arm A2 compared to the high screening intensity arm A1 at the ages of 25 and 28 (Figure 2; Table S1) was respectively 1.03 (95% CI, 0.54–1.99) and 0.97 (95% CI, 0.50–1.88) approximating the null hypothesis. Excluding all participants negative for HPV16/18 at age 18 had no material effect on these point estimates 1.03 (95% CI, 0.53–1.98) and 0.96 (95% CI, 0.50–1.87).

## 4 DISCUSSION

The occurrence of histologically confirmed HSIL cases in the high‐intensity and low‐intensity screening arms was almost equivalent. Notably vaccine‐targeted HPV types 16/18 and vaccine‐cross‐protected HPV31/45 had very low prevalence and were absent from the HSIL cases throughout the 6 years of follow‐up (8 to 16 years post‐vaccination).

The similarity of HSIL findings in the low screening intensity arm as compared to the high screening intensity arm in women vaccinated up to 16 years earlier has important public health implications. First of all, this is the first trial evidence to support cervical screening that focuses only on immediate cervical cancer precursor lesions in women who received HPV vaccination as early adolescents. Our results underline that among vaccinated women a reduction of unnecessary screening that identifies indifferent cytological findings is as safe and effective as high‐intensity screening conventionally applied in unvaccinated women.

Almost all the HSIL cases were caused by non‐vaccine‐covered HPV types. These are known to be less oncogenic but have been found present in cancer cases caused by multiple HPV infections.^22^ Apart from HPV33‐associated HSIL, the majority of HSIL cases among HPV‐vaccinated women probably have low potential to progress into cancer as recently demonstrated by the lack of hypermethylation of cervical cancer risk genes in HSIL.^23^

A moderate increase in repeated ASCUS lesions of borderline statistical significance was noted in the low screening intensity arm at all trial visits. However, this was not reflected in the frequency of HSIL. Furthermore, the overall prevalence of cytological HSIL and LSIL findings did not differ between the two screened arms.

The relatively high numbers of LSIL cases deserve to be noted, especially considering the high prevalence of non‐vaccine targeted high‐risk HPV types still present. The most prevalent HPV types at all screening ages and arms were HPV51 and HPV52. HPV52 belongs to species clade alpha‐9 of the high‐risk HPV types, which have shown to be partially cross‐protected by HPV16/18 vaccination.^24^ However, their prevalence and the prevalence of associated LSIL cases remained relatively high. HPV52 associated HSIL cases seem to lack indicators of progression potential.^25^ As for the rest of the identified HPV types belonging to clade alpha‐7 of the high‐risk HPV types, not only HPV16/18 vaccine‐induced cross‐protection but also the progression potential of associated LSIL is also questionable.^26^ Taken together our trial results imply that in vaccinated women LSIL findings but also repeated ASCUS findings are probably better left untreated or not diagnosed in the first place. Implementing our trial‐based evidence will help to improve the balance between risks and benefits of cytological screening in countries with established HPV vaccination programs. The strengths of our study are its population‐based nature and the systematic allocation trial design. The high compliance of participants throughout the three study visits makes our active follow‐up reliable. The HPV vaccination of participants in the arms A1 and A2 took place well before the average age of sexual debut in Finland (16.5 years), approximately age 14 (12 to 15) similar to current HPV vaccination programs.

This trial has two main limitations: the allocation into high‐intensity or low‐intensity screening by even an odd birth date instead of randomization, and the use of cytology as the primary screening methodology. In principle, the birth date‐based allocation results in homogeneous distributions of participant characteristics in a random fashion. On the other hand, total openness, had it materialized, would have introduced potential bias between trial arms,^27^ for example, in participation or in possible opportunistic cervical screening visits. Our trial was blinded one‐way, as the screening results, except for HSIL+, were concealed from the low screening intensity arm participants, and both recruitment and overtime participation were identical between the trial arms (Appendix S1) indicating a lack of known biases. Eventually, however, randomization would have tackled possible unknown biases between the trial arms. Cytology, instead of HPV genotyping, was used as the primary screening method due to the early start of the trial more than 10 years ago. HPV‐based screening has since then been proven more efficacious than cytology‐based screening due to its higher sensitivity, reproducibility, and possibility for automatization.^26^, ^28^ This is now allowing a longer screening interval among unvaccinated women. Our results suggest that the HPV‐based screening interval among vaccinated women can be even longer.

Screening trials among HPV vaccinated women have been scarce.^17^, ^29^ Both the immunity to the most high‐risk HPV types and the changed HPV type distribution as a result of vaccination are now causing a decline in the PPV of screening as cervical lesions are decreasing in vaccinated populations, especially for women vaccinated at younger ages.^12^, ^30^, ^31^ Therefore, the frequency of any screening might need to be revisited in order to have the most cost‐effective strategy and avoid harms related to screening and subsequent possible treatment for precancerous lesions.^10^, ^11^, ^12^, ^32^

Among unvaccinated women, primary HPV screening has been shown to result in an increased detection of HSIL/CIN2+ during follow‐up, with longer screening intervals being shown to be safe in comparison to primary cytology‐based screening.^10^, ^32^, ^33^, ^34^, ^35^, ^36^ Thus, HPV screening permits longer screening intervals further reducing the harms of too frequent screening in vaccinated women. The number needed to screen, and the number needed to follow up to prevent one cancer case have been seen to vary substantially among HPV types and were higher in low‐oncogenicity types in the pre‐vaccination era.^37^ The striking reduction in HPV16/18 observed in our findings (88% reduction by age 28) supports the idea that high intensity of screening and treatment probably is not necessary in vaccinated women, the decision factor for low‐intensity screening being mainly the decline in HPV prevalence.

The uptake of HPV vaccination in this study was 52%. This coverage is not considered very high but resulted in the reduction of vaccine‐covered HPV types^5^, ^9^, ^16^ and shows that the setting of this trial can be extrapolated to fit many other countries, especially countries with high coverage of vaccination and established vaccination programs but also those that miss school‐based programs and have low to moderate HPV‐vaccination coverage. In conclusion, our study is the first comparative trial in HPV‐vaccinated women to assess the non‐inferiority of low‐intensity versus high‐intensity screening and provides unequivocal evidence on the safety and efficacy of less frequent cytology‐based screening against CIN2+ among HPV‐vaccinated women aged 22 to 28.

## AUTHOR CONTRIBUTIONS

**Mònica Ortega Llobet:** Visualization; investigation; writing – original draft; writing – review and editing; formal analysis; data curation; validation; methodology. **Penelope Gray:** Formal analysis; supervision; writing – review and editing; visualization; data curation; funding acquisition; methodology; resources; project administration; investigation. **Iacopo Baussano:** Methodology; writing – review and editing; funding acquisition. **K. Miriam Elfström:** Funding acquisition; writing – review and editing. **Tiina Eriksson:** Data curation; writing – review and editing; project administration; investigation. **Camilla Lagheden:** Investigation; data curation; methodology; writing – review and editing; validation; supervision. **Pekka Nieminen:** Methodology; writing – review and editing; data curation; investigation. **Anna Söderlund‐Strand:** Methodology; writing – review and editing; data curation. **Joakim Dillner:** Conceptualization; methodology; resources; writing – review and editing; funding acquisition; supervision. **Ville N. Pimenoff:** Data curation; methodology; writing – review and editing; funding acquisition; project administration. **Matti Lehtinen:** Conceptualization; resources; supervision; funding acquisition; writing – review and editing; project administration; investigation; writing – original draft; methodology.

## CONFLICT OF INTEREST STATEMENT

There are no conflicts of interest to declare. Where authors are identified as personnel of the International Agency for Research on Cancer/World Health Organization, the authors alone are responsible for the views expressed in this article and they do not necessarily represent the decisions, policy or views of the International Agency for Research on Cancer /World Health Organization.

## ETHICS STATEMENT

This trial (NCT02149030) was approved by the ethical review board of the Pirkanmaa Hospital District, Tampere, Finland in 2013. The HPV vaccination trial (PI ML) has pertinent, ethical review board clearances (Tampere University Hospital/PIRHA Ethical Review Board), registry‐linkage permissions (THL, Valvira) and data protection (FinData) approvals for the trial registered at clinicaltrialsgov.com (NCT02149030). Participants' informed consents cover pertinent health registry linkages, most notably Finnish Cancer Registry linkages and retrieval of diagnostic histopathological blocks from biobanks and pathology laboratories.

## Supporting information


**Table S1.** Hazard ratios of HSIL/CIN2+ and respective 95% confidence intervals (CI) by screening visit of low‐intensity versus high‐intensity screening arms (A2/A1) and of safety screening arm versus high‐intensity screening arm (A3/A1) calculated per protocol for: (A) All participants as and (B) only participants negative for HPV16/18.
**Appendix S1.** The numbers and relative proportions of participants allocated and attending the trial at the respective ages of 22, 25, and 28 years by trial arm and vaccinated birth cohort.

## Data Availability

R code is publicly available on GitHub (https://github.com/Monicaortegallobet/finnishscreeningtrial). Anonymous study data is available from the corresponding author upon reasonable request after approval by DAC. The raw data from this study is available upon request from the DAC and ethical approval via https://etsin.fairdata.fi.

## References

[1] IARC Working Group on the Evaluation of Carcinogenic Risks to Humans. & International Agency for Research on Cancer . Human Papillomaviruses, Vol 70, World Health Organization, International Agency for Research on Cancer, Lyon 2007.

[2] European Medicines Agency . Gardasil. 2006 https://www.ema.europa.eu/en/medicines/human/EPAR/gardasil

[3] European Medicines Agency . Cervarix. https://www.ema.europa.eu/en/medicines/human/EPAR/cervarix 2007.

[4] Sinka K , Kavanagh K , Gordon R , et al. Achieving high and equitable coverage of adolescent HPV vaccine in Scotland. J Epidemiol Community Health. 2014;68:57‐63. 23986492 10.1136/jech-2013-202620

[5] Lehtinen M , Luostarinen T , Vanska S , et al. Gender‐neutral vaccination provides improved control of human papillomavirus types 18/31/33/35 through herd immunity: results of a community randomized trial (III). Int J Cancer. 2018;143:2299‐2310. 29845626 10.1002/ijc.31618

[6] Palmer T , Wallace L , Pollock KG , et al. Prevalence of cervical disease at age 20 after immunisation with bivalent HPV vaccine at age 12‐13 in Scotland: retrospective population study. BMJ Open. 2019;365:l1161. doi:10.1136/bmj.l1161 PMC6446188 30944092

[7] Thamsborg LH , Napolitano G , Larsen LG , Lynge E . Impact of HPV vaccination on outcome of cervical cytology screening in Denmark—a register‐based cohort study. Int J Cancer. 2018;143:1662‐1670. 29707775 10.1002/ijc.31568 PMC6175001

[8] Lei J , Ploner A , Elfstrom KM , et al. HPV vaccination and the risk of invasive cervical cancer. N Engl J Med. 2020;383:1340‐1348. 32997908 10.1056/NEJMoa1917338

[9] Lehtinen M , Soderlund A , Vanska S , et al. Impact of gender‐neutral or girls‐only vaccination against human papillomavirus—results of a community‐randomized clinical trial (I). Int J Cancer. 2018;142:949‐958. 29055031 10.1002/ijc.31119

[10] Ronco G , Dillner J , Elfstrom KM , et al. Efficacy of HPV‐based screening for prevention of invasive cervical cancer: follow‐up of four European randomized controlled trials. Lancet. 2014;383:524‐532. 24192252 10.1016/S0140-6736(13)62218-7

[11] El‐Zein M , Richardson L , Franco EL . Cervical cancer screening of HPV vaccinated populations: cytology, molecular testing, both or none. J Clin Virol. 2016;76:S62‐S68. doi:10.1016/j.jcv.2015.11.020 26631958 PMC4789074

[12] Lei J , Ploner A , Lehtinen M , et al. Impact of HPV vaccination on cervical screening performance: a population‐based cohort study. Br J Cancer. 2020;123:156‐160. 10.1038/s41416-020-0850-6 PMC7341799 32362659

[13] Gray P , Kann H , Pimenoff V , et al. Human papillomavirus seroprevalence in pregnant women following gender‐neutral and girls‐only vaccination programs in Finland: a cross‐sectional cohort analysis following a cluster randomized trial. PLoS Med. 2021;18:1‐18. 10.1371/journal.pmed.1003588 PMC8216524 34097688

[14] Drolet M , Bernard E , Boilly M‐C , et al. Population‐level impact and herd effects following human papillomavirus vaccination programmes: a systematic review and meta‐analysis. Lancet Infect Dis. 2015;15:565‐580. 25744474 10.1016/S1473-3099(14)71073-4 PMC5144106

[15] Cuschieri K , Palmer T , Graham C , Cameron R , Roy K . The changing nature of HPV associated with high grade cervical lesions in vaccinated populations, a retrospective study of over 1700 cases in Scotland. Br J Cancer. 2023;129:1134‐1141. doi:10.1038/s41416-023-02386-9 37563221 PMC10539290

[16] Pimenoff VN , Gray P , Louvanto K , et al. Ecological diversity profiles of non‐vaccine‐targeted HPVs after gender‐based community vaccination efforts. Cell Host Microbe. 2023;31:1921‐1929. 37944494 10.1016/j.chom.2023.10.001

[17] Louvanto K , Eriksson T , Elfstrom M , et al. Baseline findings and safety of infrequent vs. frequent screening of human papillomavirus vaccinated women. Int J Cancer. 2020;147:440‐447. 31749143 10.1002/ijc.32802

[18] Lehtinen M , Baussano I , Apter D , et al. Characteristics of a cluster‐randomized phase IV human papillomavirus vaccination effectiveness trial. Vaccine. 2015;33:1284‐1290. 25593103 10.1016/j.vaccine.2014.12.019

[19] Söderlund‐Strand A , Dillner J , Carlson J . High‐throughput genotyping of oncogenic human papilloma viruses with MALDI‐TOF mass spectrometry. Clin Chem. 2008;54:86‐92. 17981923 10.1373/clinchem.2007.092627

[20] Söderlund‐Strand A , Carlson J , Dillner J . Modified general primer PCR system for sensitive detection of multiple types of oncogenic human papillomavirus. J Clin Microbiol. 2009;47:541‐546. 19144817 10.1128/JCM.02007-08 PMC2650955

[21] Ejegod DM , Junge K , Franzmann M , et al. Clinical and analytical performance of the BD OnclarityTM HPV assay for detection of CIN2+ lesions on SurePath samples. Papillomavirus Res. 2016;2:31‐37. 29074183 10.1016/j.pvr.2016.01.003 PMC5886872

[22] Pimenoff VN , Tous S , Benavente Y , et al. Distinct geographic clustering of oncogenic human papillomaviruses multiple infections in cervical cancers: results from a worldwide cross‐sectional study. Int J Cancer. 2019;144:2478‐2488. 30387873 10.1002/ijc.31964

[23] Louvanto K , Verhoef L , Pimenoff V , et al. Low methylation marker levels among human papillomavirus‐vaccinated women with cervical high‐grade squamous intraepithelial lesions. Int J Cancer. 2024;155:1549‐1557. doi:10.1002/ijc.35044 38801336

[24] Wheeler CM , Castellsagué X , Garland SM , et al. Cross‐protective efficacy of HPV‐16/18 AS04‐adjuvanted vaccine against cervical infection and precancer caused by non‐vaccine oncogenic HPV types: 4‐year end‐of‐study analysis of the randomised, double‐blind PATRICIA trial. Lancet Oncol. 2012;13(1):100‐110. doi:10.1016/S1470-2045(11)70287-X 22075170

[25] Petäjä T , Keranen H , Karppa T , et al. Immunogenicity and safety of human papillomavirus (HPV)‐16/18 AS04‐adjuvanted vaccine in healthy boys aged 10–18 years. J Adolesc Health. 2009;44:33‐40. 19101456 10.1016/j.jadohealth.2008.10.002

[26] Kann H , Hortlund M , Eklund C , Dillner J , Faust H . Human papillomavirus types in cervical dysplasia among young HPV‐vaccinated women: population‐based nested case–control study. Int J Cancer. 2020;146(9):2539‐2546. doi:10.1002/ijc.32848 31868230

[27] Altman DG , Bland JM . Treatment allocation in controlled trials: why randomize? BMJ. 1999;318:1209. 10221955 10.1136/bmj.318.7192.1209 PMC1115595

[28] Naucler P , Ryd W , Törnberg S , et al. Human papillomavirus and Papanicolaou tests to screen for cervical cancer. N Engl J Med. 2007;357:1589‐1597. 17942872 10.1056/NEJMoa073204

[29] Bhatia R , Kavanagh K , Cubie HA , et al. Use of HPV testing for cervical screening in vaccinated women—insights from the SHEVa (Scottish HPV prevalence in vaccinated women) study. Int J Cancer. 2016;138:2922‐2931. 26845632 10.1002/ijc.30030

[30] Franco EL , Cuzick J , Hildesheim A , de Sanjosé S . Issues in planning cervical cancer screening in the era of HPV vaccination. Vaccine. 2006;24S:171‐177. 10.1016/j.vaccine.2006.05.061 16844268

[31] Palmer TJ , MacFadden M , Pllock KGJ , et al. HPV immunization and cervical screening‐confirmation of changed performance of cytology as a screening test in immunized women: a retrospective population‐based cohort study. Br J Cancer. 2016;114:582‐589. 26931370 10.1038/bjc.2015.474 PMC4782203

[32] Dillner J , Rebolj M , Pirembaut P , et al. Long term predictive values of cytology and human papillomavirus testing in cervical cancer screening: joint European cohort study. BMJ. 2008;337:a1754. doi:10.1136/bmj.a1754 18852164 PMC2658827

[33] Arbyn M , Ronco G , Anttila A , et al. Evidence regarding human papillomavirus testing in secondary prevention of cervical cancer. Vaccine. 2012;30S:88‐99. 10.1016/j.vaccine.2012.06.095 23199969

[34] Meijer CJLM , Berkhof J , Castle PE , et al. Guidelines for human papillomavirus DNA test requirements for primary cervical cancer screening in women 30 years and older. Int J Cancer. 2009;124:516‐520. 18973271 10.1002/ijc.24010 PMC2789446

[35] Aoyama‐Kikawa S , Fujita H , Hanley SJB , et al. Comparison of human papillomavirus genotyping and cytology triage, COMPACT study: design, methods and baseline results in 14 642 women. Cancer Sci. 2018;109:2003‐2012. 29660849 10.1111/cas.13608 PMC5989866

[36] Nordqvist Kleppe S , Andersson H , Elfström KM , Dillner J . Evaluation of co‐testing with cytology and human papillomavirus testing in cervical screening. Prev Med. 2023;166:107364. doi:10.1016/j.ypmed.2022.107364 36435231

[37] Wang J , Elfström KM , Lagheden C , et al. Impact of cervical screening by human papillomavirus genotype: population‐based estimations. PLoS Med. 2023;20(10):e1004304. doi:10.1371/journal.pmed.1004304 37889928 PMC10637721

