# Supplementary material for: Controlled trial of cervical cancer screening frequency among human‐papillomavirus‐vaccinated women
Source: Int J Cancer. 2025 Nov 7;158(7):1941–51. doi: 10.1002/ijc.70229 (PMC12875181; doi:10.1002/ijc.70229)
Supplement: Supplementary file 1 — Table S1. Hazard ratios of HSIL/CIN2+ and respective 95% confidence intervals (CI) by screening visit of low‐intensity versus high‐intensity screening arms (A2/A1) and of safety screening arm versus high‐intensity screening arm (A3/A1) calculated per protocol for: (A) All participants as and (B) only participants negative for HPV16/18. Appendix S1. The numbers and relative proportions of participants allocated and attending the trial at the respective ages of 22, 25, and 28 years by trial arm and vaccinated birth cohort. [file IJC-158-1941-s001.pdf]

Controlled trial of cervical cancer screening frequency among HPV vaccinated women

Monica Ortega Llobet; Penelope Gray, Iacopo Baussano; Miriam K., Elfström; Tiina Eriksson; Camilla Lagheden; Pekka Nieminen; Anna Söderlund-Strand; Joakim Dillner; Ville N., Pimenoff; Matti Lehtinen

Supplementary material

<u>Table of contents</u>

- Table S1
- Appendix

Table S1. Hazard ratios of HSIL/CIN2+ and respective 95% confidence intervals (CI) by screening visit of low-intensity versus high-intensity screening arms (A2/A1) and of safety screening arm versus high-intensity screening arm (A3/A1) calculated per protocol for: A) All participants as and B) only participants negative for HPV16/18.

A) All participants

	Hazard ratios (CI) (95% confidence intervals)
**Age of screening visit**	**A2/A1**
**25**	1.03 (0.54-1.99)
**28**	0.97 (0.50-1.88)

B) ***All participants negative for HPV16/18 at 18 years old***

	Hazard ratios (CI)
	**A2/A1**
**25**	1.03 (0.53-1.98)
**28**	0.96 (0.50-1.87)

Appendix. The numbers and relative proportions of participants allocated and attending the trial at the respective ages of 22, and 25 and 28 years by trial arm and vaccinated birth cohort.

	**Number by trial arm**		**Ratio**	**Ratio***
**Screening visits by birth cohort**	**A1**	**A2**	**A2/A1**	**A2/A1**
Visit 1 (Age 22)				
**1992**	619	670	1.08	1.04
**1993**	719	739	1.03	0.99
**1994**	741	785	1.06	1.02
**1995**	660	693	1.05	1.01
**Total**	2740	2886	1.05	1.01
Visit 2 (Age 25)				
**1992**	551	603	1.09	1.05
**1993**	634	667	1.05	1.01
**1994**	674	733	1.09	1.09
**1995**	606	648	1.07	1.03
**Total**	2465	2651	1.08	1.04
Visit 3 (Age 28)				
**1992**	511	574	1.12	1.08
**1993**	592	625	1.06	1.02
**1994**	634	683	1.08	1.04
**1995**	563	599	1.06	1.02
**Total**	2300	2481	1.08	1.04
**Total all visits**	7505	8018	1.07	1,03

*Corrected by ratio of odd/even birthdays (745/716=1.04) of 1992-95 born infrequent (A2) and frequent (A1) screening arm participants
